# Effects of Thermal Denaturation on the Interactions Between Soluble Soybean Polysaccharides and Casein and Whey Protein

**DOI:** 10.3390/molecules30214207

**Published:** 2025-10-28

**Authors:** Hongyang Pan, Seng Zhou, Xiaofang Chu, Zhaojun Wang, Jie Chen

**Affiliations:** 1 State Key Laboratory of Food Science and Resources, Jiangnan University, Wuxi 214122, China; 2 Analysis and Testing Center, Jiangnan University, Wuxi 214122, China; 3 Institute of Botany, Jiangsu Province and Chinese Academy of Sciences, Nanjing 210014, China

**Keywords:** soluble soybean polysaccharides, interactions, thermal denaturation, casein, centrifugal sedimentation rate, LUMisizer stability

## Abstract

This study aimed to investigate the interactions between soluble soybean polysaccharides (SSPS) and milk proteins, namely, casein and whey protein, and to evaluate their effects on the stability of acidified milk beverages under different degrees of thermal denaturation. Casein, whey protein, and SSPS were used as raw materials to prepare mixed solutions under varying pH conditions. A combination of analytical techniques, including centrifugal sedimentation rate, particle size distribution, ζ-potential measurement, differential scanning calorimetry (DSC), size-exclusion chromatography (SEC), and LUMisizer stability analysis, was employed to systematically examine the interactions between SSPS and the two proteins, as well as the influence of thermal treatment at 120–140 °C (casein) and 65–78 °C (whey protein). The results demonstrated that under acidic conditions (pH 3.5–4.5), SSPS formed compact complexes with casein, effectively stabilizing casein dispersions through steric hindrance and electrostatic repulsion. In contrast, SSPS exhibited a limited stabilizing ability toward whey protein due to its strong tendency to aggregate, which hindered the formation of uniform complexes. Regarding thermal denaturation, casein heated at 140 °C for more than 40 min showed pronounced κ-casein dissociation and aggregation, resulting in reduced stability of the SSPS–casein system. For whey protein, increasing thermal denaturation (complete denaturation at 78 °C for 30 min) led to the formation of larger aggregates, with particle size increasing from 198.23 nm to 213.33 nm and ζ-potential decreasing from −3.77 mV to −2.01 mV, thereby diminishing the stability of the SSPS–whey protein system. Overall, this study elucidates the interaction mechanisms of SSPS with casein and whey protein, and highlights the role of thermal denaturation, thereby providing theoretical guidance for the effective application of SSPS in acidified milk beverages.

## 1. Introduction

Acidified milk beverages are popular among consumers for their refreshing taste and nutritional value, yet their stability remains a major challenge that limits industrial development. Under acidic conditions near the isoelectric point, milk proteins tend to aggregate and precipitate, thus requiring the addition of stabilizers to suppress this process. Pectin, a commonly used stabilizer, maintains long-term stability of milk proteins mainly through electrostatic repulsion along its galacturonic acid backbone. In contrast, soluble soybean polysaccharide (SSPS), a novel natural polysaccharide with a molecular composition and structure similar to those of pectin, confers short-term stabilization primarily through steric hindrance arising from neutral sugar side chains such as arabinose and galactose. This mechanistic difference endows SSPS with unique application potential in acidified dairy systems [1,2,3].

In bovine milk proteins, casein constitutes 80–82% and has an isoelectric point of pH 4.6 with strong thermal stability, as denaturation begins at approximately 120 °C [4]. Whey proteins account for about 20%, with an isoelectric point of pH 5.1–5.3 and relatively poor thermal stability, as denaturation occurs above 65 °C [5,6]. In industrial production, raw materials for acidified milk beverages, such as fresh milk or low-denatured skim milk powder, are commonly subjected to thermal processes including pasteurization and spray-drying, which may induce partial denaturation of milk proteins. Nevertheless, the effect of such thermal denaturation on the interactions between SSPS and milk proteins has not yet been clarified.

Recent studies have provided valuable insights into the interaction mechanisms between whey proteins and soluble soybean polysaccharides (SSPS). For instance, Igartúa et al. [7] reported that heat treatment significantly affected the complexation behavior of whey protein isolate (WPI) with SSPS, altering particle size, ζ-potential, and interfacial stability. In another study, Igartúa et al. [8] demonstrated that pH, protein-to-polysaccharide ratio, and ionic strength critically influence the electrostatic complexation between WPI and SSPS. Similarly, Zamani et al. [9] confirmed that SSPS can effectively stabilize acidified whey protein systems through steric and electrostatic repulsion mechanisms.

However, these studies mainly focused on whey protein–SSPS interactions, while the corresponding behavior of casein–SSPS systems remains poorly understood. Moreover, how thermal denaturation of these proteins modulates their interactions with SSPS has not yet been systematically clarified. Understanding these distinct interaction mechanisms is essential for optimizing the formulation and processing of acidified dairy beverages stabilized by natural polysaccharides.

At present, substantial gaps remain in understanding the interactions between SSPS and milk proteins. First, the specific contributions of casein and whey protein to SSPS–protein interactions have not been clearly defined. Second, limited information is available regarding the regulatory effect of thermal denaturation on these interactions and on the stability of the systems [3,10]. These knowledge gaps restrict the precise and effective application of SSPS in acidified milk beverages.

Therefore, this study aims to fill these gaps by systematically investigating how thermal denaturation of milk proteins affects their interactions with SSPS. By comparing SSPS–casein and SSPS–whey protein systems before and after controlled heat treatment, this work elucidates the underlying mechanisms governing complex formation, aggregation behavior, and stability under thermal stress. The findings provide new insights into optimizing the formulation and thermal processing of SSPS-stabilized acidified dairy beverages.

Building on this background, the present study investigates the specific interactions of SSPS with casein and whey proteins, elucidates the underlying mechanisms under different pH conditions, and further examines the influence of thermal denaturation on these interactions and on system stability. The findings not only enhance the theoretical understanding of polysaccharide–protein interactions but also provide scientific guidance for formulation optimization and process improvement in acidified milk beverages, thereby supporting the efficient application of natural polysaccharide stabilizers.

## 2. Results and Discussion

### 2.1. Interaction Between SSPS and Casein Under Acidic Conditions

At 20 °C, the isoelectric point of casein is pH 4.6, while SSPS exhibits minimal net charge around pH 3.2. The interactions were investigated at pH 3.5, 3.8, 4.0, 4.2, 4.5, and 4.8 using centrifugal sedimentation rate, particle size, and LUMisizer stability as evaluation indices.

**Figure 1:**
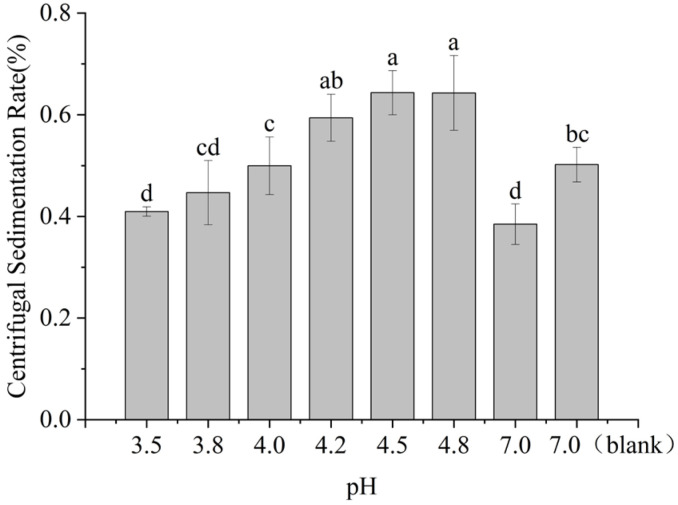
Effect of SSPS on centrifugal sedimentation rate of 1.0% casein solution at different pH conditions. (7.0 (blank) refers to a 1.0% casein solution at pH 7.0 without the addition of SSPS). Different letters indicate significant differences among samples (*p* < 0.05).

**Figure 2:**
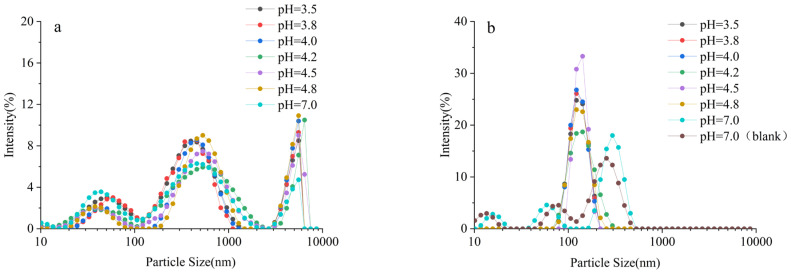
Particle size distribution of SSPS and SSPS-casein complex at different pH conditions. (**a**) SSPS, (**b**) SSPS-casein complex. (pH 7.0 (blank) refers to a 1.0% casein solution without the addition of SSPS).

As shown in Figure 1 and Figure 2, when 0.4% SSPS was present alone, the particle size distribution was highly polydisperse. Under neutral conditions (pH 7.0), mixing with casein eliminated the polydispersity, and the particle size became comparable to that of casein, indicating weak interactions between the two. SSPS loosely adsorbed on the surface of casein, slightly increasing the complex size while improving stability.

As the pH decreased from 7.0 to 4.5, the particle size of casein alone increased markedly, resulting in sedimentation. In contrast, the particle size of the SSPS–casein mixture decreased and became more uniform, accompanied by a lower sedimentation rate (Table 1). This effect can be attributed to the fact that, at lower pH, casein transitions from negatively charged to nearly neutral, leading to micelle dissociation and κ-casein collapse, which reduces particle size. SSPS remains negatively charged, enhancing its interaction with casein to form uniform complexes, and the long side chains of SSPS provide steric hindrance that effectively stabilizes casein.

As the pH decreased from 4.5 to 3.5, both the sedimentation rate and particle size of the system decreased, while the polydispersity index remained nearly unchanged. Under these conditions, casein changed from electrically neutral to positively charged, which enhanced its interaction with negatively charged SSPS, leading to tighter binding and improved stability. Previous studies have shown that when pH < 5, casein micelles transform from spherical to chain-like structures, thereby increasing the surface area of the “dissipative layer”, which enlarges the contact area between SSPS and the micelles and strengthens electrostatic adsorption [11].

**Figure 3:**
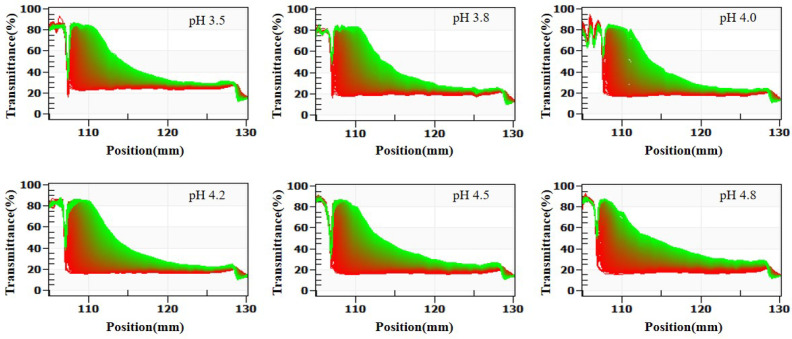
Effect of SSPS on LUMisizer stabilization of 1.0% casein solution at different pH conditions. (Green regions indicate stable dispersions with low sedimentation, whereas red regions correspond to faster sedimentation or less stable areas).

In summary, SSPS stabilizes casein primarily through steric hindrance at pH 4.5–7.0. At lower pH values (3.2–4.5), both steric hindrance and electrostatic repulsion contribute, resulting in superior stability. This conclusion is supported by LUMisizer stability analysis (Figure 3), where samples at pH 3.5–4.8 (except pH 4.8) showed good stability after centrifugation. In the figure, green regions indicate stable dispersions with low sedimentation, whereas red regions correspond to faster sedimentation or less stable areas, highlighting the potential for long-term stability.

### 2.2. Interaction Between SSPS and Whey Protein Under Acidic Conditions

**Figure 4:**
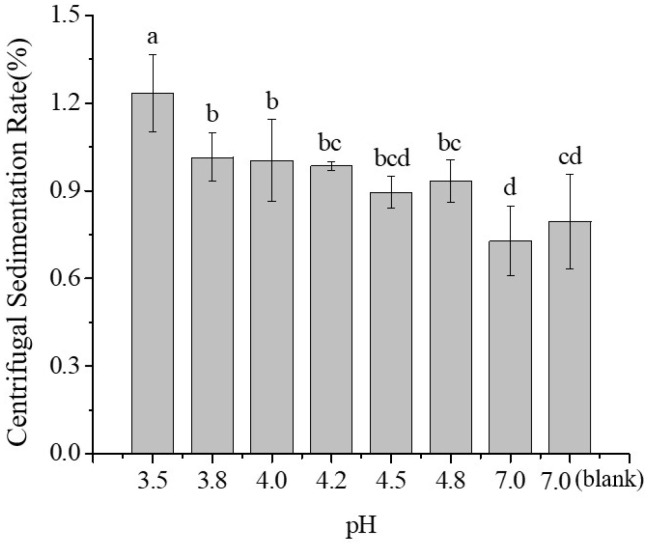
Effect of SSPS on centrifugal sedimentation rate and particle size of 1.0% whey protein solution at different pH conditions. (At pH 7.0, “blank” refers to a 1.0% whey protein solution without the addition of SSPS). Different letters indicate significant differences among samples (*p* < 0.05).

**Figure 5:**
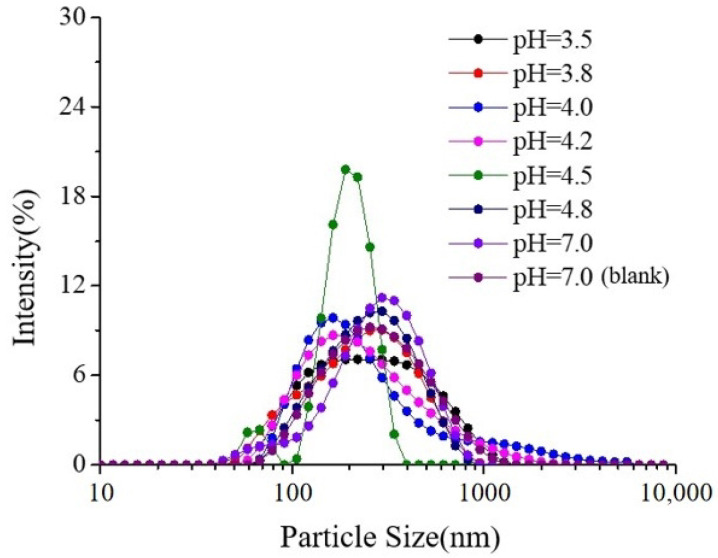
Effect of SSPS on particle size distribution of 1.0% whey protein solution at different pH conditions. (At pH 7.0, “blank” refers to a 1.0% whey protein solution without the addition of SSPS).

The sedimentation rate, particle size distribution, and related parameters of SSPS–whey protein interactions are shown in Figure 4 and Figure 5 and Table 2. When whey protein was present alone at pH 7.0, the particle size was large and stability was poor. Although food-grade concentrated whey protein undergoes low heat treatment, it exhibits a high degree of denaturation [12,13,14,15], resulting in numerous aggregates and a broad molecular weight distribution. Under neutral conditions, the addition of SSPS improved stability. The intrinsic polydispersity of SSPS disappeared, and the particle size matched that of whey protein, indicating weak interactions between the two. SSPS loosely adsorbed onto the surface of whey protein aggregates but did not alter the complex particle size, likely because the aggregates themselves were relatively large [3,16].

As the pH of whey protein decreased, the stability of the system in the range of pH 4.8–3.8 was poor. After mixing with SSPS, stability improved at pH 3.5–4.8; however, the sedimentation rate remained relatively high and slightly increased as pH decreased, while particle size and polydispersity index changed only minimally (Table 2). The isoelectric point of whey protein is pH 5.1–5.3, and below this pH, whey protein carries a positive charge. Although the positive charge increased with decreasing pH, the stability of the SSPS–whey protein mixture was not significantly enhanced, likely due to the abundance of whey protein aggregates, which hindered the formation of uniform complexes with SSPS. Although the average particle size of whey protein increased as pH decreased, the polydispersity index (PDI) remained relatively unchanged (0.26–0.28). This is likely due to the presence of abundant whey protein aggregates, which dominate the system and limit the formation of uniform complexes with SSPS. As a result, the overall width of the particle size distribution does not increase significantly, despite changes in average size.

**Figure 6:**
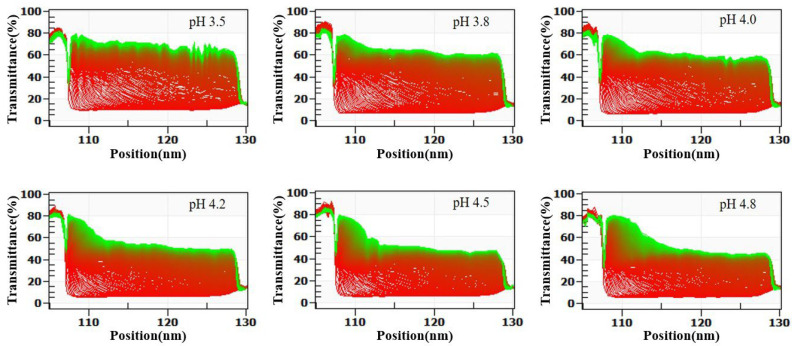
Effect of SSPS on LUMisizer stabilization of 1.0% whey protein solution at different pH conditions. (Green regions indicate stable dispersions with low sedimentation, whereas red regions correspond to faster sedimentation or less stable areas).

The LUMisizer stability results in Figure 6 are consistent with those shown in Figure 4. With decreasing pH, the stability of the SSPS–whey protein mixtures progressively declined. Overall, within the pH range of 3.5–4.8, SSPS provided only short-term stabilization of whey protein.

### 2.3. Interaction Between SSPS and Thermally Denatured Casein Under Acidic Conditions

**Figure 7:**
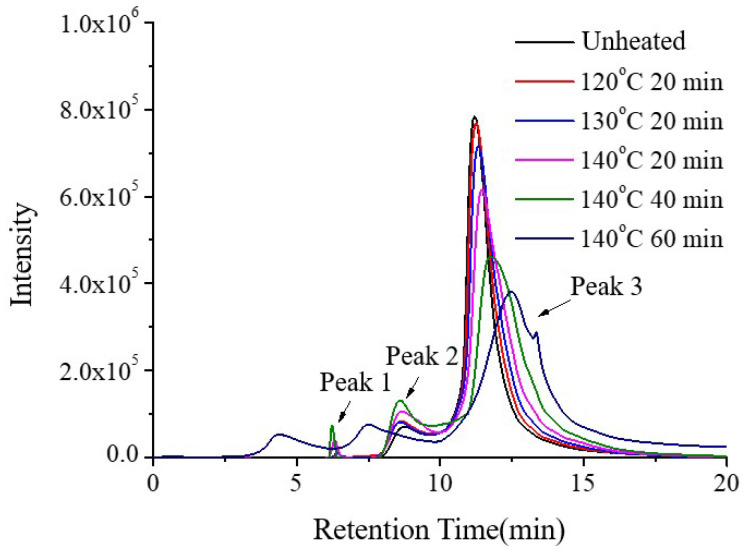
Effect of heat treatment of casein on the SEC elution profiles.

It has been reported that casein exhibits high thermal stability, undergoing denaturation between 120 °C and 140 °C. Using unheated casein as the control, the molecular weight distribution of the soluble fraction of casein was analyzed after heating at 120 °C and 130 °C for 20 min, and at 140 °C for 20, 40, and 60 min. As shown in Figure 7, Peak 3 (Mw: 13.9 kDa) represents the major component of casein. Due to heating, κ-casein on the surface of casein dissociates. With increasing heating intensity, the degree of κ-casein dissociation gradually increases, causing Peak 3 to shift to higher retention times, which corresponds to a decrease in the molecular weight of the main casein fraction, while the proportion of Peak 2 (Mw: 481 kDa) gradually increases. Heating at 130 °C for 20 induced the formation of new soluble aggregates, represented by Peak 1 (Mw: 8209 kDa). When heated at 140 °C for 60 min, two larger soluble aggregates were observed. These results indicate that with increasing thermal treatment, casein polypeptide chains unfold and protein molecules aggregate, resulting in an overall increase in molecular weight.

**Figure 8:**
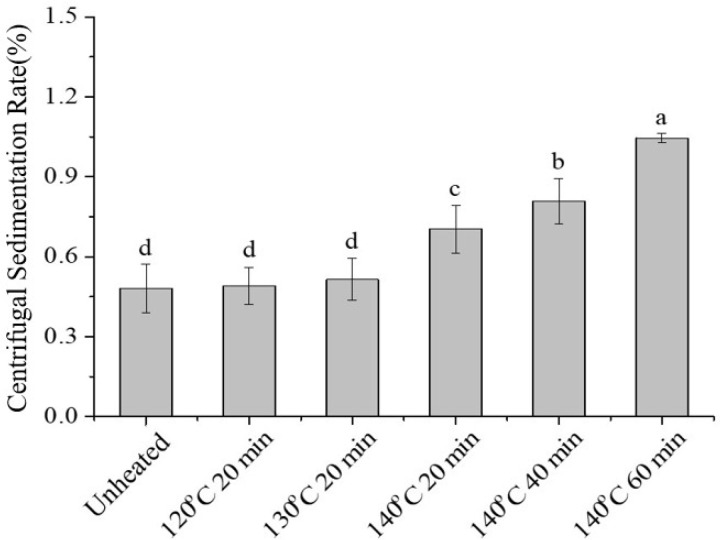
Effect of heat treatment on the average particle size and polydispersity index of SSPS–casein mixed solutions. Different letters indicate significant differences among samples (*p* < 0.05).

During the production of skimmed milk powder, processes such as preheating and spray drying involve high temperatures, which may induce partial thermal denaturation of casein. Therefore, in this study, the effect of thermally treated casein on the stability of SSPS–casein mixtures at pH 4.0 with 0.4% SSPS as a stabilizer was investigated. As shown in Figure 8, heating casein at 120 °C or 130 °C for 20 min had no significant effect on the sedimentation rate of the mixture (*p* > 0.05). However, heating at 140 °C resulted in a gradual increase in sedimentation rate with longer treatment durations.

Casein undergoes thermal denaturation within the range of 120–140 °C. As shown in Figure 7, Peak 3 (Mw: 13.9 kDa) represents the major component of casein. Upon heating, κ-casein dissociated, and with increasing thermal intensity, Peak 3 shifted to higher retention times (corresponding to a decrease in molecular weight), while the proportion of Peak 2 (Mw: 481 kDa) increased. Heating at 130 °C for 20 min led to the appearance of a new aggregate, Peak 1 (Mw: 8209 kDa), and at 140 °C for 60 min, even larger aggregates were observed, indicating unfolding of casein polypeptide chains and aggregation resulting in increased molecular weight.

The particle size of the SSPS–casein mixture (Table 3) initially decreased and then increased with increasing heating intensity. Heating at 140 °C for 20 min, dominated by κ-casein dissociation, led to smaller particle size, whereas heating for more than 40 min promoted aggregation, resulting in larger particle size. The polydispersity index increased significantly at 140 °C for 40 min (approximately five times that of the unheated sample), indicating a broader particle size distribution. In addition, ζ-potential decreased with increasing heating, indicating weakened electrostatic repulsion.

Figure 8 shows that at pH 4, heating casein at 120 °C or 130 °C for 20 min had no significant effect on the sedimentation rate of the mixture (*p* > 0.05), while at 140 °C, the sedimentation rate increased with prolonged heating time, consistent with the observed aggregation and particle size changes.

**Figure 9:**
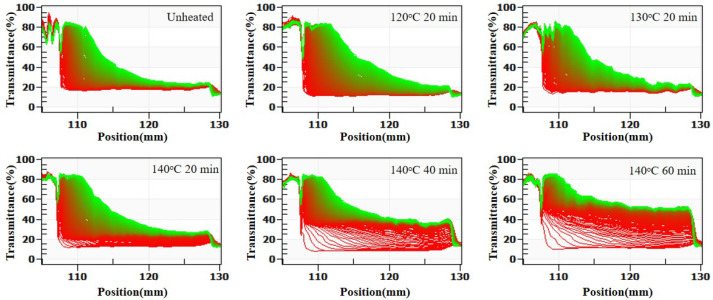
Effect of heat treatment of casein on LUMisizer stabilization of SSPS and casein solution. (Green regions indicate stable dispersions with low sedimentation, whereas red regions correspond to faster sedimentation or less stable areas).

Figure 9 (LUMisizer stability) demonstrates that stability decreased with increasing heating. When casein was heated at 140 °C for more than 40 min, the initial transmittance after centrifugation rose sharply due to the sedimentation of large aggregates, and the dissociation of κ-casein increased turbidity, reducing transmittance to 40–60%. In summary, heating at 120 °C or 130 °C for 20 min had little effect on system stability, whereas heating at 140 °C for over 40 min severely compromised stability.

### 2.4. Interaction Between SSPS and Thermally Denatured Whey Protein Under Acidic Conditions

**Figure 10:**
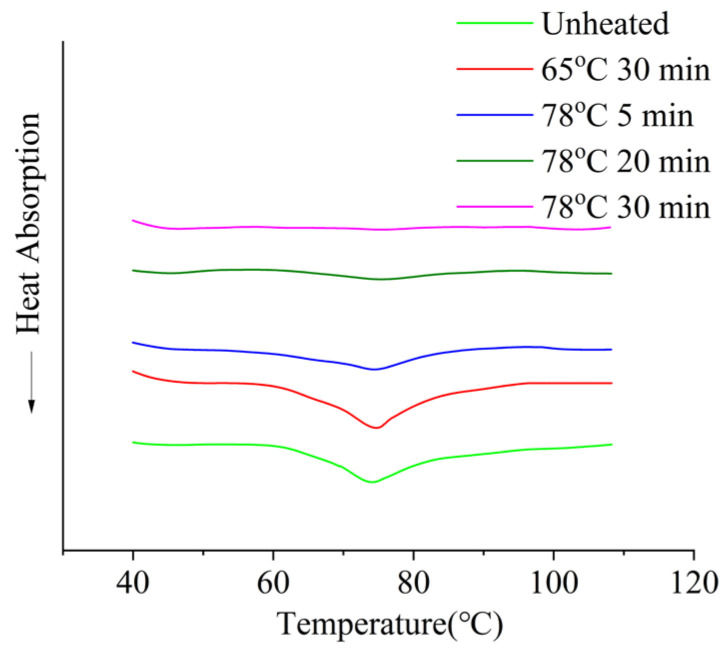
Effect of heat treatment on the DSC cure of whey protein.

Whey protein exhibits relatively low thermal stability. β-lactoglobulin is heat-labile, beginning to denature at 65–70 °C, whereas α-lactalbumin denatures above 70 °C [13,17]. As shown in Figure 10 (DSC curves) and Table 4, the degree of whey protein denaturation increased with more intense heating, and complete denaturation was achieved after treatment at 78 °C for 30 min.

**Figure 11:**
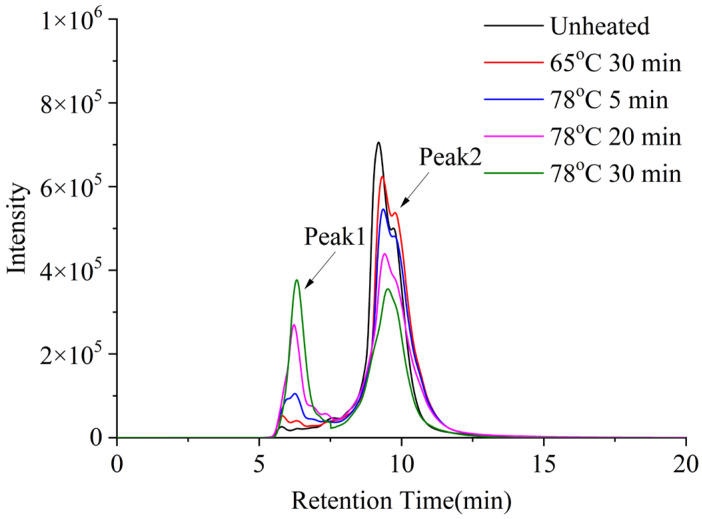
Effect of heat treatment of whey protein on the SEC elution profiles.

As shown in Figure 11 (molecular weight distribution), Peak 1 (Mw: 33.3 kDa) represents the major component of unheated whey protein, while Peak 2 (Mw: 45.3 kDa) corresponds to large aggregates. With increasing heating intensity, the proportion of Peak 1 decreased and that of Peak 2 increased, indicating progressive aggregation of whey protein [18].

**Figure 12:**
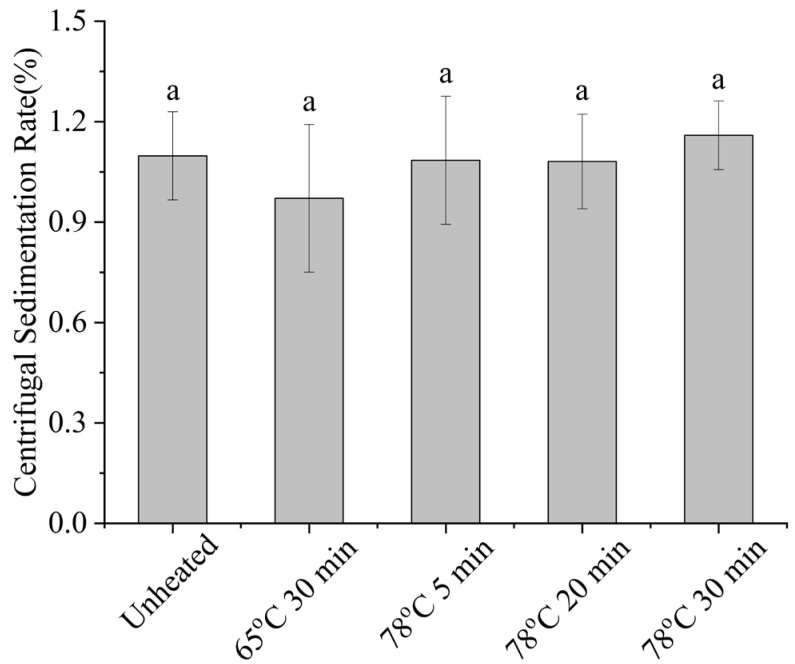
Effect of heat treatment of whey protein on the centrifugal sedimentation rate of SSPS and whey protein solution. Different letters indicate significant differences among samples (*p* < 0.05).

**Figure 13:**
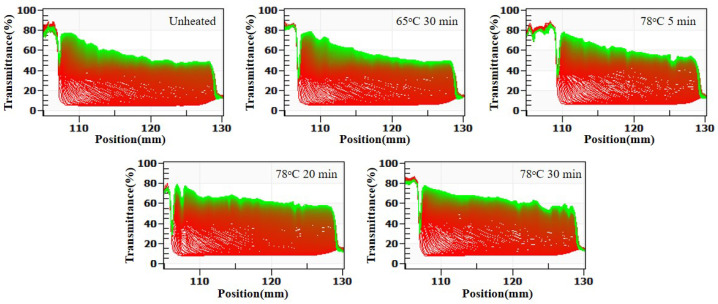
Effect of heat treatment of whey protein on LUMisizer stabilization of SSPS and whey protein solution. (Green regions indicate stable dispersions with low sedimentation, whereas red regions correspond to faster sedimentation or less stable areas).

As shown in Figure 12 (centrifugal sedimentation rate) and Table 5, at pH 4, increasing thermal denaturation of whey protein had no significant effect on the sedimentation rate or polydispersity index, but particle size increased and ζ-potential decreased. Thermal denaturation led to the enlargement of whey protein aggregates, and the positive charge below the isoelectric point increased. Consequently, the negative charge of SSPS–whey protein complexes decreased, weakening electrostatic repulsion. The LUMisizer stability results (Figure 13) were consistent with Table 5, showing that the stability of the system gradually decreased with increasing whey protein denaturation.

## 3. Materials and Methods

### 3.1. Materials and Reagents

Soluble soybean polysaccharides (SSPS, CA 700) were purchased from Fuji Oil Co., Ltd. (Osaka, Japan). Casein and whey protein isolate (protein content ≥ 90%) were obtained from Fonterra Co-operative Group Ltd. (Auckland, New Zealand). Citric acid, sodium citrate, copper sulfate, potassium tartrate, sodium hydroxide, sodium carbonate, and other analytical grade chemicals were purchased from Sinopharm Chemical Reagent Co., Ltd. (Shanghai, China).

### 3.2. Instruments and Equipment

A high-pressure homogenizer (AH-BASIC, Lerong’en Technology Co., Ltd., Shanghai, China), a refrigerated centrifuge (SIGMA 3K15, Sigma, Osterode am Harz, Germany), a laser particle size analyzer (Nano-ZS, Malvern Instruments, Malvern, UK), and a stability analyzer (LUMisizer, LUM GmbH, Berlin, Germany) were used in this study. An ultrapure water purification system (EPED Technology Co., Ltd., Nanjing, China), a thermostatic drying oven (HY300, Yiheng Scientific Instruments Co., Ltd., Shanghai, China), a high-performance liquid chromatograph (Alliance 2695, Waters, Milford, MA, USA), and a differential scanning calorimeter (Q100, TA Instruments, New Castle, DE, USA) were also employed.

### 3.3. Preparation of Milk Protein–SSPS Mixed Solutions

An accurately weighed amount of casein (or whey protein) was dispersed in deionized water and stirred at room temperature (25 °C) for 1 h to ensure complete hydration. SSPS was dissolved separately in deionized water at 70 °C and stirred on a magnetic stirrer at the same temperature for 30 min until fully hydrated. After both solutions were cooled to room temperature, they were combined and stirred for 10 min. The pH of the mixture was then carefully adjusted using 30% citric acid solution while stirring at room temperature for another 10 min to achieve uniform acidification. The mixed solution was subsequently heated to 60 °C and homogenized at a pressure of 200 bar. After homogenization, the mixture was heated in a 65 °C water bath for 30 min to obtain the final product. The final mixtures contained 1.0% (*w*/*v*) protein and 0.4% (*w*/*v*) SSPS.

### 3.4. Thermal Treatment of Milk Protein Solutions

An accurately weighed amount of casein (or whey protein) was dispersed in deionized water to obtain a protein concentration of 1.0% (*w*/*v*), followed by stirring at room temperature for 1 h to ensure complete hydration. According to the thermal denaturation range of casein, the solutions were heated at 120 °C and 130 °C for 20 min, and at 140 °C for 20, 40, and 60 min, respectively. For whey protein, based on its denaturation range, the solutions were heated at 65 °C for 30 min and at 78 °C for 5, 20, and 30 min to achieve different degrees of denaturation. All thermal treatments were conducted in closed glass tubes placed in a thermostatic water bath, and the temperature was maintained within ±0.2 °C using a calibrated digital thermometer. Each treatment was performed in triplicate to ensure reproducibility. Untreated casein (or whey protein) solutions were used as the control.

### 3.5. Differential Scanning Calorimetry (DSC)

The degree of denaturation of whey protein samples subjected to different heating conditions was determined using a differential scanning calorimeter (Q100, TA Instruments, USA) [19]. Accurately weighed samples (10–15 mg) were sealed in aluminum pans with lids. Thermal scans were performed from 40 °C to 110 °C at a heating rate of 5 °C/min. The onset denaturation temperature (T_0_), peak denaturation temperature (T_p_), and denaturation enthalpy (ΔH) were obtained using Universal Analysis 2000 software. The degree of denaturation was calculated according to the following equation:

$$Degree\;of\;Denaturation\;\left(\%\right)=\frac{\Delta H_{C}-\Delta H_{t}}{\Delta H_{C}}$$

In this equation, ΔH_c_ denotes the denaturation enthalpy of untreated whey protein, whereas ΔH_t_ denotes the denaturation enthalpy of whey protein subjected to different heat treatments.

### 3.6. Determination of Molecular Weight Distribution

The molecular weight distribution of casein and whey protein was analyzed using a high-performance liquid chromatography system (Shimadzu, Kyoto, Japan) equipped with a KW-804 protein column (Shodex, Tokyo, Japan) and a KW-802 protein column (Shodex, Tokyo, Japan). Detection was carried out with a UV detector at 220 nm. The column temperature was maintained at 30 °C, and the injection volume was 10 μL. The mobile phase consisted of 0.05 mol/L phosphate buffer (pH 7.0) containing 0.3 mol/L sodium chloride, with a flow rate of 1.0 mL/min.

### 3.7. Determination of Centrifugal Sedimentation Rate

A clean 10 mL centrifuge tube was weighed (m_0_), and 8 mL of acidified milk beverage was added, followed by weighing again (m_1_). The sample was centrifuged at 7000 r/min for 10 min at 4 °C. Following centrifugation, the supernatant was discarded, the tube was inverted for 5 min to drain residual liquid, and then weighed (m_2_). The sedimentation rate was calculated as follows [20]:

$$Sedimentation\;Rate\;\left(\%\right)=\frac{m_{2}-m_{0}}{m_{1}-m_{0}}\times 100$$

### 3.8. Determination of Particle Size and ζ-Potential

The particle size distribution and ζ-potential of milk proteins in acidified milk beverages were measured using a Malvern laser particle size analyzer. Measurement parameters were set as follows: sample refractive index, 1.590; water refractive index, 1.330; and test temperature, 25 °C [21]. To avoid multiple scattering caused by high sample concentration, the beverages were diluted 1:100 with 0.02 mol/L citrate buffer adjusted to the same pH as the original samples.

### 3.9. LUMisizer Stability Analysis

Acidified milk beverages were centrifuged at 4 °C at 7000× *g*, 10,000× *g*, 15,000× *g*, and 25,000× *g* for 1 h and 2 h, respectively, to separate the system into supernatant and sediment. The supernatant was filtered through a 0.45 μm membrane and analyzed for polysaccharide content using gel permeation chromatography (GPC) [22]. In GPC, separation was carried out on a TSK-GEL-5000PWXL column (7.8 mm × 300 mm, Tosoh Corporation, Tokyo, Japan) with a differential refractive index (RI) detector and a UV detector (Shimadzu, Kyoto, Japan) on a high-performance liquid chromatography system. The RI and UV signals were used to quantify polysaccharide and protein contents in the supernatant. The column temperature was maintained at 40 °C, the injection volume was 30 μL, and the mobile phase consisted 0.1 mol/L phosphate buffer (pH 6.8) at a flow rate of 0.6 mL/min.

For quantification, standard curves were constructed with SSPS and pectin concentrations plotted on the *x*-axis and peak area on the *y*-axis. The standard curve for SSPS was y = 326,886x (R^2^ = 0.9990), and that for pectin was y = 307,373x (R^2^ = 0.9995).

### 3.10. Data Analysis

All experiments were performed at least in triplicate. Results are expressed as mean ± standard deviation. Data were analyzed by one-way ANOVA with Tukey’s test using SPSS 2.0, with significance at *p* < 0.05.

## 4. Conclusions

This study systematically investigated the interactions between soluble soybean polysaccharide (SSPS) and milk proteins, focusing on the effects of thermal denaturation. SSPS formed stable complexes with casein under acidic conditions, providing stabilization through a combination of steric hindrance and electrostatic repulsion, whereas its stabilizing effect on whey protein was limited, resulting in only short-term stabilization. The most stable SSPS–casein complexes were observed at pH 3.5–4.5 with minimal thermal denaturation, while for whey protein, SSPS provided only short-term stabilization under similar conditions. Thermal denaturation influenced these interactions, with prolonged heating promoting κ-casein dissociation and aggregation, leading to reduced stability in SSPS–casein systems, while increasing whey protein denaturation favored large aggregate formation and decreased stability. Overall, SSPS showed stronger stabilizing effects on casein than on whey protein, and the degree of thermal denaturation is a key factor regulating these interactions.

These findings provide a theoretical basis for the application of SSPS in acidified dairy beverages and for optimizing product stability through controlled thermal treatment. Future research could explore the effects of varying SSPS concentrations, other polysaccharides, and application in real beverage systems to further enhance stabilization.

## Figures and Tables

**Table 1:** Effect of SSPS on the average particle size and polydispersity index of 1.0% casein solution at different pH conditions.

pH (1.0% Casein Solution)	Average Particle Size (nm)	Polydispersity Index	pH (1.0% Casein + 0.4% SSPS Mixed Solution)	Average Particle Size (nm)	Polydispersity Index
3.5	70.32 ± 0.75 ^b^	0.40 ± 0.01 ^b^	3.5	123.90 ± 0.49 ^b^	0.06 ± 0.01 ^bc^
3.8	Flocculation		3.8	126.07 ± 0.54 ^b^	0.05 ± 0.01 ^bc^
4.0	Extensive Precipitation		4.0	132.37 ± 2.38 ^b^	0.04 ± 0.02 ^c^
4.2	Extensive Precipitation		4.2	135.10 ± 0.16 ^b^	0.08 ± 0.01 ^b^
4.5	Extensive Precipitation		4.5	138.43 ± 2.21 ^b^	0.07 ± 0.02 ^bc^
4.8	626.17 ± 108.58 ^a^	0.61 ± 0.07 ^a^	4.8	129.70 ± 0.22 ^b^	0.08 ± 0.00 ^b^
7.0	132.87 ± 9.44 ^b^	0.57 ± 0.03 ^a^	7.0	228.20 ± 25.70 ^a^	0.36 ± 0.02 ^a^

Different letters within the same column indicate significant differences between the data (*p* < 0.05).

**Table 2:** Effect of SSPS on the average particle size and polydispersity index of 1.0% whey protein solution at different pH conditions.

pH (1.0% Casein Solution)	Average Particle Size (nm)	Polydispersity Index	pH (1.0% Casein + 0.4% SSPS Mixed Solution)	Average Particle Size (nm)	Polydispersity Index
3.5	229.63 ± 4.39 ^b^	0.27 ± 0.01 ^b^	3.5	208.97 ± 0.26 ^c^	0.28 ± 0.00 ^a^
3.8	Flocculation		3.8	201.07 ± 3.29 ^d^	0.26 ± 0.01 ^b^
4.0	Flocculation		4.0	200.37 ± 0.85 ^d^	0.26 ± 0.01 ^b^
4.2	Flocculation		4.2	196.93 ± 3.54 ^d^	0.26 ± 0.00 ^b^
4.5	Flocculation		4.5	186.63 ± 2.64 ^e^	0.22 ± 0.01 ^c^
4.8	694.43 ± 8.49 ^a^	0.49 ± 0.03 ^a^	4.8	219.30 ± 5.35 ^b^	0.21 ± 0.01 ^c^
7.0	229.93 ± 2.52 ^b^	0.26 ± 0.01 ^b^	7.0	244.70 ± 1.90 ^a^	0.27 ± 0.00 ^a^

Different letters within the same column indicate significant differences between the data (*p* < 0.05).

**Table 3:** Effect of heat treatment of casein on the average particle size and polydispersity index of SSPS and casein solution.

Thermal Treatment Conditions	Average Particle Size (nm)	Polydispersity Index	ζ-Potential (mV)
Unheated	137.55 ± 2.45 ^bc^	0.05 ± 0.01 ^e^	−2.17 ± 0.44 ^b^
120 °C 20 min	135.60 ± 2.59 ^bc^	0.08 ± 0.01 ^d^	−1.93 ± 0.23 ^b^
130 °C 20 min	134.85 ± 1.05 ^bc^	0.09 ± 0.00 ^cd^	−1.74 ± 0.26 ^b^
140 °C 20 min	130.90 ± 2.80 ^c^	0.11 ± 0.00 ^c^	−0.85 ± 0.36 ^a^
140 °C 40 min	143.35 ± 1.55 ^ab^	0.24 ± 0.00 ^b^	−0.82 ± 0.06 ^a^
140 °C 60 min	153.75 ± 3.25 ^a^	0.36 ± 0.00 ^a^	−0.67 ± 0.18 ^a^

Different letters within the same column indicate significant differences between the data (*p* < 0.05).

**Table 4:** Effect of heat treatment on the denaturation degree of whey protein.

Thermal Treatment Conditions	Degree of Denaturation
Unheated	0
65 °C 30 min	8.74
78 °C 5 min	49.58
78 °C 20 min	84.55
78 °C 30 min	100

**Table 5:** Effect of heat treatment of whey protein on the average particle size and polydispersity index of SSPS and whey protein solution.

Thermal Treatment Conditions	Average Particle Size (nm)	Polydispersity Index	ζ-Potential (mV)
Unheated	198.23 ± 1.82 ^b^	0.25 ± 0.03 ^b^	−3.77 ± 0.14 ^d^
65 °C 30 min	202.07 ± 4.48 ^ab^	0.28 ± 0.01 ^a^	−3.15 ± 0.37 ^c^
78 °C 5 min	219.57 ± 3.27 ^a^	0.28 ± 0.00 ^a^	−2.52 ± 0.27 ^b^
78 °C 20 min	219.60 ± 1.72 ^a^	0.26 ± 0.01 ^ab^	−2.37 ± 0.27 ^ab^
78 °C 30 min	213.33 ± 0.83 ^ab^	0.26 ± 0.01 ^ab^	−2.01 ± 0.06 ^a^

Different letters within the same column indicate significant differences between the data (*p* < 0.05).

## Data Availability

Data are contained within the article.
